# Death of American Physicians

**Published:** 1854-11

**Authors:** 


### DEATH OF AMERICAN PHYSICIANS.

We regret to announce the death of two eminent members of our profession within a brief space. Dr. Waldo I. Burnett, the distinguished microscopist, died, in Boston, a few weeks since, and we have received the intelligence of the death of Dr. Swett, in New York. Dr. Swett stood among the foremost in his profession, for his skill in pathology and physical diagnosis, and, like Dr. Burnett, was known as an author, and admired for his pure devotion to science. The early death of these zealous cultivators of medical science is a public loss. They promised to shed lustre upon the medical profession of their country.

